# Evaluating the Nutritional Composition of Unripe Citrus and Its Effect on Inhibiting Adipogenesis and Adipocyte Differentiation

**DOI:** 10.4014/jmb.2403.03015

**Published:** 2024-04-19

**Authors:** Sunghee Kim, Eunbi Lee, Juhye Park, Ju-Ock Nam, Soo Rin Kim

**Affiliations:** 1 Research Institute of Tailored Food Technology, Kyungpook National University, Daegu 41566, Republic of Korea; 2 School of Food Science and Biotechnology, Kyungpook National University, Daegu 41566, Republic of Korea

**Keywords:** Unripe citrus, nutritional composition, hesperidin, adipogenesis, anti-obesity

## Abstract

Citrus fruits offer a range of health benefits due to their rich nutritional profile, including vitamin C, flavonoids, carotenoids, and fiber. It is known that unripe citrus has higher levels of vitamin C, dietary fiber, polyphenols, and flavonoids compared to mature fruits. In this study, we assessed the nutritional components of unripe citrus peel and pressed juices, as well as their anti-obesity potential through the modulation of adipocyte differentiation and the expression of adipogenesis-related genes, specifically PPARγ and C/EBPα, in 3T3-L1 preadipocytes. Our analysis revealed that unripe citrus peel exhibited elevated levels of fiber and protein compared to pressed juice, with markedly low levels of free sugar, particularly sucrose. The content of hesperidin, a representative flavonoid in citrus fruits, was 3,157.6 mg/kg in unripe citrus peel and 455.5 mg/kg in pressed juice, indicating that it was approximately seven times higher in unripe citrus peel compared to pressed juice. Moreover, we observed that the peel had a dose-dependently inhibitory effect on adipocyte differentiation, which was linked to a significant downregulation of adipogenesis-related gene expression. Thus, our findings suggest that unripe citrus possesses anti-obesity effects by impeding adipogenesis and adipocyte differentiation, with the peel demonstrating a more pronounced effect compared to pressed juice.

## Introduction

Obesity is a chronic disease characterized by excessive body fat accumulation and highlights its significance as a major risk factor for metabolic syndrome, which includes type 2 diabetes, dyslipidemia, and cardiovascular disease [1, 2]. The prevalence of obesity continues to increase worldwide, and currently, approximately one-third of the world’s population suffers from overweight or obesity [3]. Various drugs such as Orlistat, Phentermine/Topiramate, and Naltrexone/bupropion complex are used to treat and prevent obesity [4]. However, continuous use of these drugs is difficult due to side effects such as vomiting [5], steatorrhea [6, 7], insomnia, and anxiety [8]. Therefore, the development of anti-obesity functional materials derived from food or natural products rather than pharmaceuticals is attracting attention.

The development of functional materials to improve obesity is evaluated based on their ability to reduce body fat through various mechanisms, including inhibiting fat absorption, promoting fat oxidation, inhibiting fat synthesis, and controlling appetite [9-11]. Dysregulation of adipogenesis and adipocyte differentiation contributes to the development of obesity by promoting adipose tissue expansion and altering adipocyte function [12]. Adipogenesis is the process by which preadipocytes differentiate into mature adipocytes, and morphological and functional changes occur through adipocyte differentiation. During adipocyte differentiation, various transcription factors and signaling pathways are involved in adipocyte development, lipid accumulation and metabolism, including upregulation of adipogenic transcription factors such as peroxisome proliferator-activated receptor gamma (PPARγ) and CCAAT/enhancer-binding proteins (C/EBPs) [13]. Understanding the molecular mechanisms underlying adipogenesis and adipocyte differentiation is essential for developing therapeutic strategies to combat obesity and related health risks.

Citrus fruits offer a range of health benefits due to their rich nutritional profile, including vitamin C, flavonoids, carotenoids, and fiber [14]. Especially, flavonoids are known to possess physiological activities such as promoting homeostasis, exerting anti-inflammatory effects, and exhibiting anti-cancer properties [15]. Unripe citrus refers to immature fruits that are harvested and discarded every year between August and September to enhance citrus quality. Unripe citrus is also known as green tangerine because of its green peel, but it is a completely different species from green tangerine (*Citrus nippokoreana*). Unlike other tangerines, green tangerines are a variety of traditional Jeju tangerines whose skin remains green until February and ripens to yellow around March or April. Approximately 50,000 to 100,000 tons of unripe citrus are discarded each year through fruit thinning, and most of the discarded unripe citrus cause environmental pollution around orchards [16].

A significant amount of waste is generated during the production and processing of citrus fruits, including unripe citrus. Notably, about 18% of citrus production is used for juice production, resulting in waste equivalent to nearly 50% of the mass of fresh fruit. Of this waste, 30-40% consists of citrus peels, which are rich in various bioactive ingredients such as polyphenols, flavonoids, essential oils, pectin, and cellulose, leading to an increase in research focused on their utilization [17]. Also, more recently, research is being conducted to explore the replacement of expensive grain raw materials with citrus by-products in the manufacturing of animal feed and its impact on livestock growth [18]. Therefore, the utilization of citrus waste not only minimizes environmental damage but also contributes to the development of agriculture and food industries through high added value.

It is known that unripe citrus has higher levels of vitamin C, dietary fiber, polyphenols, and flavonoids compared to mature fruits [19]. The total phenol and flavonoid content of the peel of premature mandarin (*C. unchiu*) was 94.04 mg GAE/g and 43.99 mg QE/g, respectively, which was higher than that of mature mandarin (79.15 mg GEA/g and 23.51 mg QE/g). The flavonoid content of the peel from premature and mature mandarins was about ten times higher than that of the juice, with hesperidin and narirutin being detected as major components. Hesperidin is the predominant flavonoid compound found in citrus peel and has higher antioxidant activity compared to narirutin [20]. In addition, hesperidin has been demonstrated to promote weight loss in various in vivo tests and contributes to anti-obesity treatment by inhibiting cholesterol synthesis and absorption, as well as genes associated with fat production [21]. In order to utilize the functional ingredients of unripe citrus, the shipment of unripe citrus has been temporarily allowed since 2016 in accordance with the enactment of the Jeju Special Self-Governing Province Ordinance on Tangerine Production and Distribution [22]. Research is currently being conducted on producing various foods such as jelly, scones, and vinegar using unripe citrus [23-25]. However, there is a lack of research on utilizing functional ingredients such as flavonoids found in unripe citrus for food and functional materials.

In this study, we compared the nutritional components of unripe citrus peel and pressed juice. We utilized Oil Red O staining to evaluate the suppression of differentiation and assessed the downregulation of gene expression related to adipogenesis, specifically PPARγ and C/EBPα, in 3T3-L1 preadipocytes. Our aim is to verify the efficacy against obesity.

## Materials and Methods

### Materials

The unripe citrus used in this study was harvested and processed in August and was provided by Whole Foods Korea Co., Ltd. The harvested unripe citrus was washed thoroughly and then squeezed using a juicer to obtain pressed juice. The unripe citrus peel remaining after juicing was cut and dried using hot air at 60°C for 24 h. The dried peel was ground with a grinder and used in this experiment.

### General Ingredient Analysis

The general components of unripe citrus peel (dried) and pressed juice, including moisture (method 930.15), crude protein (method 2001.11), crude fat (method 2003.05), crude fiber (method 978.10), and crude ash content (method 942.05), were measured according to the standard methods of Association of Official Analytical Chemists (AOAC) [26]. The analysis was performed at the Institute of Agricultural Science, Chungnam National University. All tests were repeated twice.

### Sample Preparation for Composition Analysis

For component analysis of unripe citrus samples, 9 ml of sterilized water was added to 1 g of unripe citrus peel (dried) to make a 10% (w/w) peel extract, while the citrus pressed juice itself was used. To enhance the extraction efficiency of ingredients in unripe citrus, both 10% peel extract and pressed juice were heat-treated at 121°C for 15 min. The heat-treated samples were then centrifuged at 4000 ×*g* for 5 min to remove residue, and the supernatant was filtered through a 0.2 μm syringe filter. All samples were prepared in triplicate.

### pH and Soluble Solids (Brix)

After transferring 1.0 ml of the sample to a 1.5 ml microtube, the pH was measured using a pH meter (SevenCompact pH meter S220, Mettler Toledo, USA). Subsequently, 0.3 ml of the sample was taken, and the soluble solids (°Brix) content was measured using a saccharometer (Atago Pocket Pal-1, Atago Co. Ltd., Japan).

### Free Sugar Analysis

The glucose, fructose, and sucrose concentrations of the unripe citrus peel and pressed juice were analyzed using high-performance liquid chromatography (HPLC 1260 series, Agilent Technologies, USA) with a refractive index (RI) detector. A Rezex-ROA Organic Acid H+ column (8%, 150 mm × 4.6 mm; Phenomenex Inc., USA) was employed, with the column temperature set at 50°C. The mobile phase consisted of 0.005N H_2_SO_4_, and the flow rate was maintained at 0.6 ml/min. The obtained chromatogram was integrated and quantified using ChemStation software.

### Hesperidin and Its Derivatives Analysis

Hesperidin and its derivatives were analyzed by HPLC (Waters, Alliance 2796 Separations System) with a photodiode array detector (Waters, Alliance 2996). The column used was a Kinetex C18 100A column (5 μ, 250 mm × 4.6 mm: Phenomenex Inc.), and the column temperature was set at 30°C. The mobile phases consisted of 0.1% phosphoric acid in water (A) and 0.1% phosphoric acid in acetonitrile (B) at a flow rate 1.0 ml/min with a gradient elution carried out as follows: 15% B from 0 to 15 min, a linear gradient to 25% B from 15 to 20 min, 25%B from 20 to 30 min, a linear gradient to 35%B from 30 to 35 min, and then held at 35%B until 45min. The peaks were detected at a wavelength of 284 nm.

### Sample Preparation for Functional Analysis

To analyze the functional properties of unripe citrus, 100 ml of 10% peel extract and pressed juice were prepared. The samples were then heat-treated at 121°C for 15 min and centrifuged at 4000 rpm for 5 min to remove residues. The supernatant was filtered using a paper filter. The filtrate was freeze-dried and dissolved in dimethyl sulfoxide (DMSO) before use.

### Functional Analysis

**Cell culture.** 3T3-L1 preadipocytes were purchased at a cost from ATCC (USA). Cells were cultured in DMEM-H containing 1% antibiotic-antimycotic and 10% newborn Calf Serum (NBCS) in an atmosphere containing 5%CO_2_. When confluence reached 60-70%, cells were treated with trypsin/EDTA solution (TE; Gibco, UK) and seeded in a 6-well plate. When confluence in 6-wells reached 100%, cells were maintained for two more days to reach the post-confluency state.

**Adipogenic differentiation.** When 3T3-L1 preadipocytes reach the post-confluency state, the cells initiate adipogenic differentiation. To initiate differentiation, the medium is replaced with differentiation medium I. Differentiation medium I has the following composition: DMEM-H containing MDI (0.5 mM methylisobutylxanthine, 0.25 mM dexamethasone, 1 μg/ml insulin, 0.125 nM indomethacin (Sigma, USA)), 1% antibiotic-antimycotic, 10% fetal Bovine Serum (FBS). On the second day of differentiation, the medium is replaced with differentiation medium II. Differentiation medium II has the following composition: DMEM-H containing 1 μg/ml insulin, 1%antibiotic-antimycotic, and 10% FBS. Differentiation medium II is treated for a total of 6 days and is replaced every two days. During the differentiation experiment, NC (Preadipocytes) and WC (White adipocytes) were set as controls. Green tea extract was set as a PC (Positive control) and treated at 200 μg/ml.

**Oil red O (ORO) staining.** On the 6th day of differentiation (terminal stage of adipogenic differentiation), adipocytes were washed once with phosphate-buffered saline (PBS) and then treated with 4% paraformaldehyde (Biosesang Inc., Republic of Korea) and fixed for 1 h. Afterward, the adipocytes were washed twice with PBS and stained with 0.6% (v/v) ORO solution (Sigma) after blocking light for 30 min. After staining, the plate was washed three times with distilled water and dried overnight. To quantify the amount of product after drying, 1 ml of isopropyl alcohol is added and stirred at room temperature for 5 min. The products were aliquoted into 200 μl, and transferred to a 96-well plate. The absorbance was measured at 450 nm using a microplate reader (Tecan, Switzerland).

**Real-time reverse transcription polymerase chain reaction (RT-PCR).** On the 8th day (the last day of adipogenic differentiation), adipocytes were washed three times with PBS. Afterward, adipocytes were treated with Trizol reagent (Takara Bio) on ice to extract intracellular mRNA. The extracted mRNA was synthesized into a cDNA library using the PrimeScriptTM RT Reagent Kit (Takara Bio). For the cDNA library, standard PCR (Takara Bio) was performed in the following steps: pre-incubation (15 min at 37°C), annealing (5 min at 50°C), extension (5 min at 98°C), and cooling (4°C). The mRNA expression was measured according to the intensity of SYBR Green using the iCycleriQTM Real-Time PCR Detection System (Bio-Rad Laboratories, USA). The specific thermal cycling of RT-PCR proceeded in the following sequence: pre-incubation (1 min at 95°C), amplification (15 s at 95°C, followed by 1 min at 60°C for 39 cycles), melting (10 s at 95°C), and cooling (5 s at 72.5°C). Measured values were normalized to the expression level of β-actin and expressed as fold change to the control (WC). Every experiment was carried out in triplicate, both technically and biologically. Table 1 lists the sequences for the RT-PCR primers that were custom-made by Macrogen (Republic of Korea).

## Results and Discussion

### Analysis of General Ingredients of Unripe Citrus

The results of the general ingredients analysis of unripe citrus peel (dried) and pressed juice are shown in Table 2. The moisture content of unripe citrus peel (dried) and pressed juice was 16.13% and 94.59%, respectively. In general, the moisture content of citrus peel is known to be 86% (Korean Standard Feed Ingredients Table), but because hot-air dried unripe citrus peel was used in this test, the moisture content was lower than the known value. To compare the ingredients of unripe citrus peel and pressed juice, the contents of other ingredients were converted to dry matter, excluding moisture content.

The crude protein content of unripe citrus peel and pressed juice was 9.91% and 5.73%, respectively, with the crude protein content of peel being higher than that of pressed juice. The crude fat content of unripe citrus peel and pressed juice was 1.29% and 4.16%, respectively, with the crude fat content of pressed juice being higher than that of peel. The crude fiber content of unripe citrus peel and pressed juice was 14.06% and 0.09%, respectively, indicating almost no crude fiber in pressed juice.

The crude fiber refers to the residue excluding ash after extraction with dilute acid, dilute alkali, alcohol, and ether. The relatively high crude fiber content can indirectly show that dietary fiber is relatively rich. Dietary fiber is known to be effective in treating and preventing obesity, and the higher crude fiber content in unripe citrus peel than in pressed juice suggests that unripe citrus peel may be a potential material with anti-obesity effects [27]. The crude ash content of unripe citrus peel and pressed juice was at similar levels, with 3.87% and 3.97%, respectively.

### Physicochemical Properties of Unripe Citrus

The pH and soluble solid content of Unripe citrus peel extract (UCPE) (10%) and pressed juice are shown in Table 3. The pH of heat-treated peel extract and pressed juice was 3.47 and 2.76, respectively, which are slightly acidic. There was no difference in pH depending on the heat treatment. The soluble solids content of heat-treated peel extract and pressed juice was 5.1% Brix and 6.4% Brix, respectively, which increased with heat treatment.

The soluble solid content and pH of citrus are known to be 12-15% and 3.8-4.3, respectively [28]. As the fruit matures, the soluble solid content and pH increase, making it less sour and more sweeter. Since unripe citrus are immature fruits, they are considered to have relatively low soluble solid content and pH compared to fully ripened fruits.

### Quantification of Glucose, Fructose, and Sucrose Contents in Unripe Citrus

**Fig. 1:**
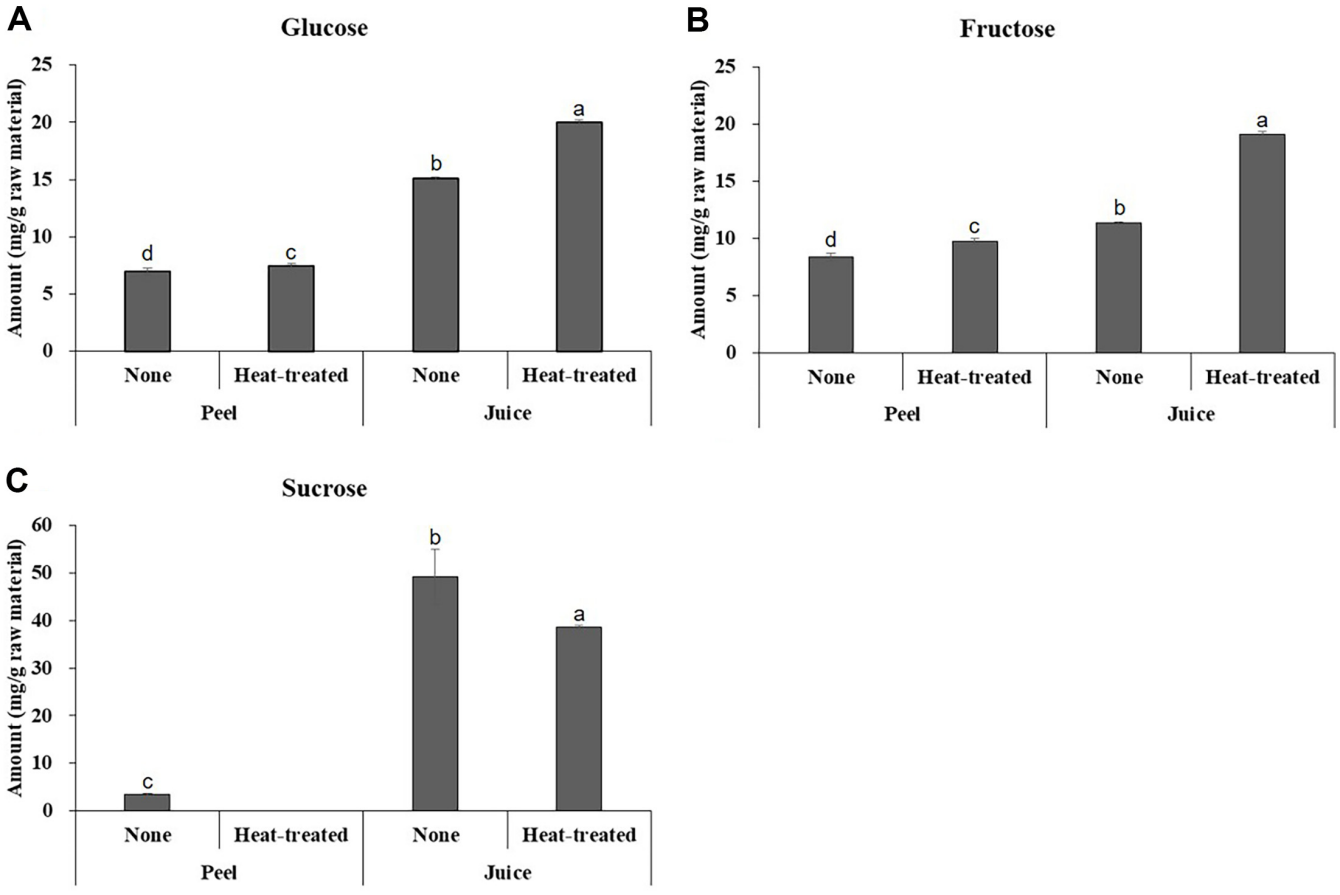
The content of glucose, fructose, and sucrose in unripe citrus peel and pressed juice. (**A**), (**B**), and (**C**) respectively show the glucose, fructose, and sucrose content. The data are presented as mean ± standard deviation, with each experiment was independently repeated in triplicate.

The glucose, fructose, and sucrose contents of unripe citrus peel and pressed juice are shown in Fig. 1. The free sugar content was expressed on a raw material basis (g). The free sugar content of unripe citrus peel(dried) was calculated by referencing the moisture content (86%) from a standard reference. The glucose and fructose content of unripe citrus peel was approximately 10 mg/g raw material, with no significant difference observed depending on heat treatment. In contrast, the sucrose content was lower than that of glucose and fructose, and it was nearly absent after heat treatment. The glucose and fructose content of unripe citrus pressed juice was similar to or higher than that of the peel, and the contents further increased with heat treatment. Additionally, the sucrose content was about ten times higher than that of the peel.

Although the soluble solids content of the heat-treated peel and pressed juice was comparable, measuring at 5.1% Brix and 6.4% Brix, respectively, the sucrose content was relatively higher in the pressed juice. Brix is used to measure sugar content, particularly sucrose content, in foods like fruits, by utilizing the relationship between the concentration of soluble solids and the refractive index. Most of the soluble solids in fruit juice are sugar, so the sugar content is measured by Brix (%). However, in addition to sugar, soluble solids include salts, amino acids, acids, pectin, etc. Therefore, a high Brix level does not necessarily indicate a high sugar content. Citrus peels, besides harboring active compounds, also contain polysaccharides, predominantly pectin, constituting 20 to 30%of their dry weight [29]. Pectin serves as a water-soluble dietary fiber, with extraction yield increasing with higher extraction temperature and time [30]. The difference in free sugar content according to the heat treatment of unripe citrus peel was minimal, but the Brix value increased by about 1.6 times, which is presumed to be due to the elution of pectin, a water-soluble component, during the heat treatment. Although there is a significant difference in sucrose content between the heat-treated peel and pressed juice, the similar Brix values are believed to be due to the effect of pectin.

Free sugars are sugars that exist in a free state rather than being bound within complex carbohydrate polymers such as starch or pectin. They include simple sugars like glucose, fructose, and sucrose. Free sugar raises blood sugar more quickly than other sugars, and continuous consumption of free sugar causes chronic diseases such as obesity and diabetes [31]. Therefore, the World Health Organization (WHO) recommends that the unnecessary intake of free sugars be reduced. The free sugar content of citrus fruits is known to continue to increase with maturity, suggesting that the utilization of immature fruits in processed citrus foods may contribute to reducing free sugar intake compared to mature fruits [32].

### Quantification of Hesperidin and Its Derivatives Contents in Unripe Citrus

In this study, the flavonoid content of unripe citrus peel and pressed juice was compared by analyzing hesperidin and its derivatives, which exhibit the highest content among citrus flavonoids. The hesperidin content of heat-treated peel and pressed juice was measured at 3,157.6 mg/kg and 455.5 mg/kg, respectively, representing an increase of approximately 6 to 7 times due to heat treatment. The content of hesperetin-7-O-glucoside, a sugar molecule bound to hesperetin, in heat-treated peel and pressed juice was 766.7 mg/kg and 75.2 mg/kg, respectively. These concentrations were approximately 4 to 6 times lower than the hesperidin content. Hesperetin, the aglycone form of hesperidin, was not detected in either sample (Table 4). The majority of flavonoids in unripe citrus are found in the glycoside form, such as hesperidin, with minimal presence of the non-glycoside form, hesperetin. The hesperidin content in unripe citrus peel was found to be approximately seven times higher than that of pressed juice, consistent with the well-established fact that flavonoid content is higher in the peel than in the pulp.

Citrus flavonoids, including narirutin, hesperidin, neohesperidin, naringin, nobiletin, and rutin, are predominantly found in the peel rather than pulp, with higher concentrations observed in unripe fruits compared to ripe ones [33, 34]. The composition and content of flavonoids vary among different citrus fruit varieties, such as grapefruit, lemon, lime, orange, and mandarin. Notably, hesperidin is the most abundant flavonoid in mandarin (*C. reticulata*). The hesperidin content of mandarin juice is known to be 24.3 mg/100 ml, which is lower than the hesperidin content found in unripe citrus pressed juice in this study (45.6 mg/100 ml). These results correspond to the fact that flavonoid content is higher in unripe fruits than in ripe fruits [35].

To compare the hesperidin content in unripe citrus peel and pressed juice according to heat treatment, we conducted hot water extraction at 121°C for 15 min. Generally, to extract hesperidin from citrus fruits, it is used by mixing methanol and ethanol or water in a certain ratio. However, the use of organic solvents such as ethanol and methanol in food is limited due to concerns regarding the toxicity of the residual solvent [36]. Extraction using water can enhance extraction efficiency through heat treatment, and this process improves the antioxidant capacity of citrus peel extract compared to untreated extract [37]. Moreover, the level of hesperidin in the pressed juice remains stable during heating or concentration treatments [38]. Therefore, heat treatment can be used to produce unripe citrus peel water extract with high flavonoid content, which can then be utilized as a functional food material to enhance health.

### Unripe Citrus Peel Extract Has a Superior Adipogenesis Inhibitory Effect Than Juice Extract

**Fig. 2:**
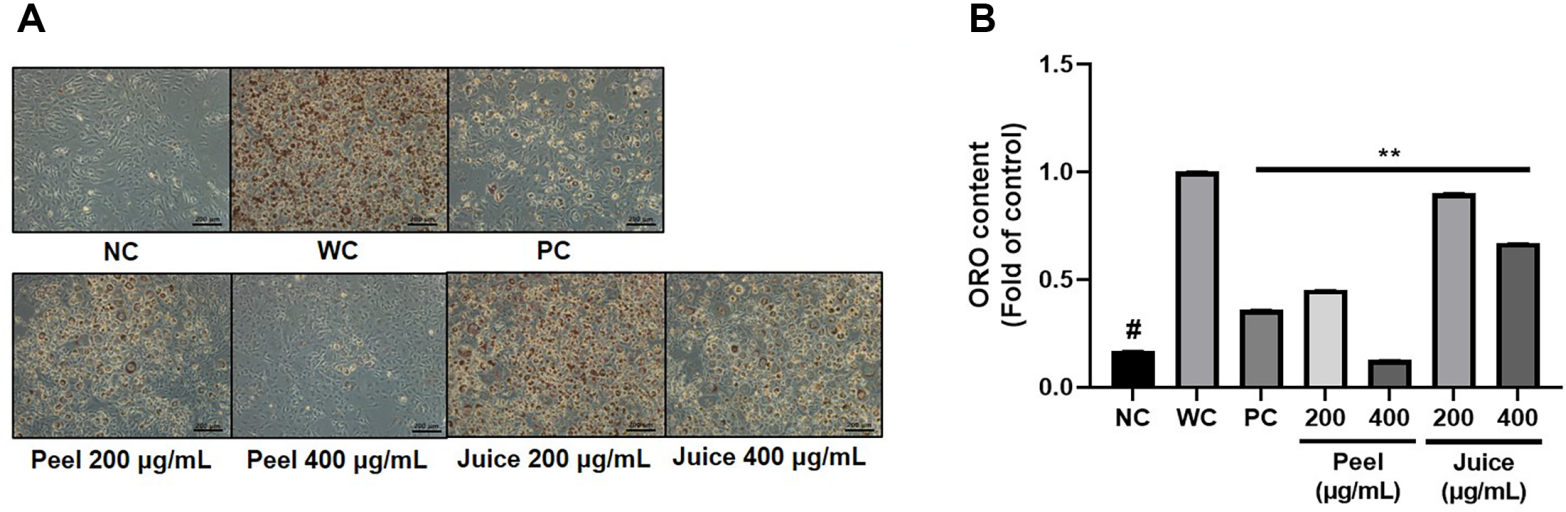
Inhibitory effect on adipogenesis of UCPE and UCJE. (**A**) Representative images of lipid droplets from each group stained with ORO solution to evaluate lipid accumulation. (**B**) After dissolving lipid droplets stained with ORO solution in isopropyl alcohol, the absorbance was measured at 450 nm. The data were presented as mean ± standard deviation, and each experiment was independently repeated in triplicate. #*p* < 0.01 for comparison between controls. **p < 0.01 for comparison between WC and experimental groups (UCPE and UCJE). NC, negative control (preadipocytes); WC, white adipocytes; PC, positive control (200 μg/ml green tea extract).

Since the process of maturation of preadipocytes into adipocytes causes adipose tissue hypertrophy, the extract's ability to inhibit adipogenic differentiation can be used as a biomarker to evaluate the anti-obesity effect. To comparison evaluation the effects of unripe citrus extract on each part, we induced mature differentiation of adipocytes for six days. Afterwards, the lipid droplets were stained and quantified. WC group, which induced differentiation without treatment, had 6.25-fold higher lipid accumulation compared to NC. UCPE treatment inhibited lipid accumulation by 55% at 200 μg/ml and 87% at 400 μg/ml. Additionally, treatment with unripe citrus juice extract (UCJE) inhibited lipid accumulation by 10% at 200 μg/ml and 33% at 400 μg/ml. Both extracts significantly inhibited adipogenesis and showed a dose-dependent effect. What is noteworthy here is that the adipogenesis inhibition effect in UCPE is 1.99 times higher at 200 μg/ml and 5.25 times higher at 400 μg/ml than in UCJE. UCPE has a similar or higher effect on inhibiting lipid accumulation than PC in the dose range of 200 – 400 μg/ml (Fig. 2A and 2B). Through the following results, we confirmed that UCPE has a better effect on inhibiting adipogenic differentiation and lipid accumulation than UCJE.

### Unripe Citrus Peel Extract Alleviated the Expression of Adipogenesis-Related Genes More Than Juice Extract

**Fig. 3:**
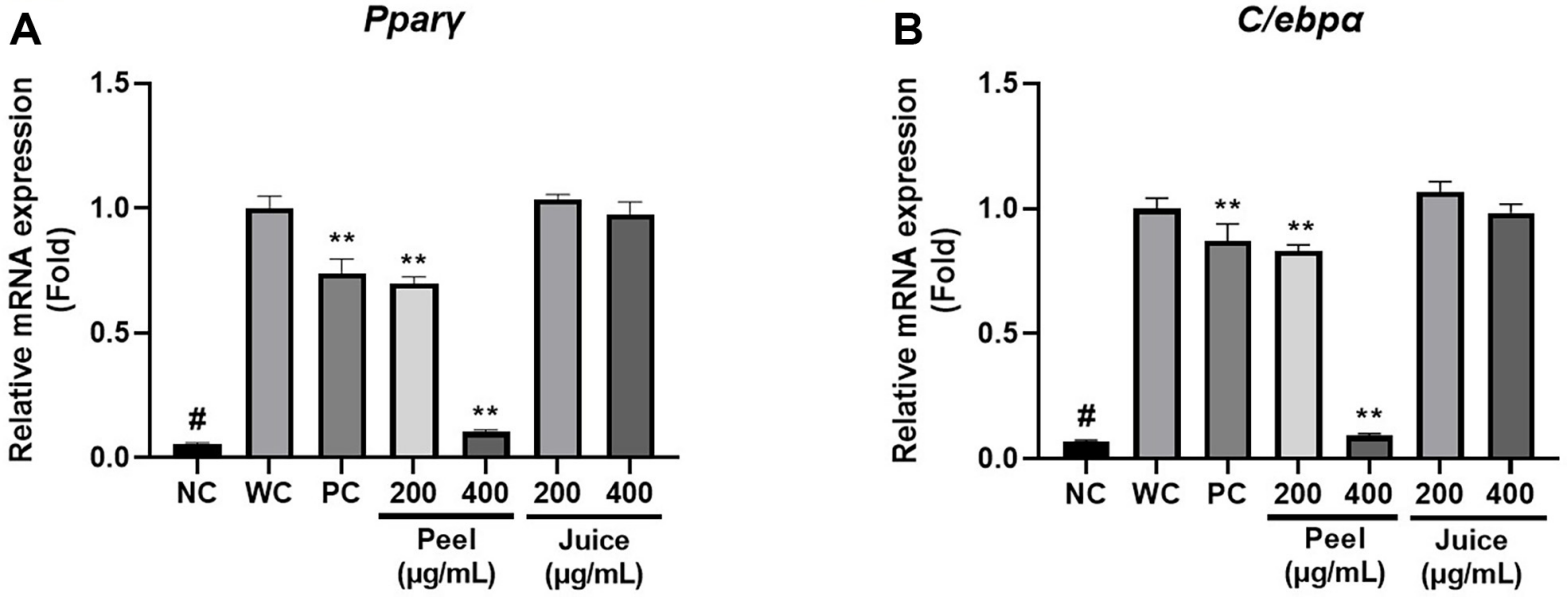
Inhibitory effect of UCPE and UCJE on the expression of adipogenesis-related genes. (**A**) Relative mRNA expression of Pparγ in 3T3-L1 adipocytes. (**B**) Relative mRNA expression of C/ebpα in 3T3-L1 adipocytes. The expression of Pparγ and C/ebpα was normalized to the expression of β-actin. The data were presented as mean ± standard deviation, and each experiment was independently repeated in triplicate. #*p* < 0.01 for comparison between controls. **p < 0.01 for comparison between WC and experimental group. NC, negative control (preadipocytes); WC, white adipocytes; PC, positive control (200 μg/ml green tea extract).

This section suggests that UCPE has a better inhibitory effect on adipogenesis-related gene expression than UCJE. Adipogenesis is a developmental process in which preadipocytes differentiate into mature adipocytes and accumulate lipids. This process is accompanied by a highly regulated and coordinated cascade of transcription factors. PPARγ and C/EBPα are the most important regulators in adipogenesis [39]. PPARγ initiates adipogenic differentiation, and genes activated by PPARγ stimulate lipid absorption and lipogenesis in adipocytes [40]. C/EBPα also promotes adipogenic differentiation by activating adipocyte-specific genes required for fatty acid synthesis, absorption, and storage [41]. Therefore, we selected PPARγ and C/EBPα as genes to evaluate adipogenesis inhibitory effect. Compared with NC, differentiation induction led to a very high level of Pparγ and C/ebpα expression in WC. There was no significant decrease in the expression of Pparγ and C/ebpα by UCJE treatment. However, when treated with UCPE, there was a significant decrease in the expression of Pparγ and C/ebpα. UCPE treatment inhibited the expression of Pparγ by 30% at 200 μg/ml and 90% at 400 μg/ml, and C/ebpα was inhibited by 17% at 200 μg/ml and 91% at 400 μg/ml. This shows a similar or higher effect of suppressing transcription factor expression when compared to PC. The inhibitory effect of UCPE on adipogenesis-related genes is dose-dependent, and the trends for both Pparγ and C/ebpα are consistent (Fig. 3A and 3B). Through the following results, we confirmed that UCPE is more effective than UCJE in suppressing adipogenesis-related gene expression.

When designing this experiment, we referred to research showing that unripe citrus fruit can have excellent anti-obesity effects. Satsuma mandarin orange (*C. unshiu*) belongs to the same genus as the unripe citrus used in this study. According to research findings, the juice and peel of green satsuma mandarin orange inhibit adipogenic differentiation and adipose tissue expansion through the leptin-PPARγ/FAS pathway, leading to a reduction in lipid accumulation [42, 43]. Based on these references, we hypothesized that unripe citrus may possess a potent anti-obesity effect. Our study analyzed biomarkers for adipose tissue reduction functionality to explore unripe citrus as a functional material for anti-obesity effects. As a result, our study suggests that the peel of unripe citrus has superior anti-obesity effects compared to its juice.

## Conclusion

Unripe citrus is generally not consumed much on its own due to its strong sour and tangy taste, but it is widely utilized for functional exploration as it contains higher levels of nutrients, such as vitamin C, dietary fiber, and potassium, compared to tangerines. Therefore, standardization of the harvesting time and usage areas of unripe citrus is a crucial factor in the exploration and efficient commercialization of anti-obesity functional materials. In this study, we evaluated the components and anti-obesity efficacy of unripe citrus peel and pressed juice. Our analysis of the composition of unripe citrus revealed higher levels of healthy fiber and protein in the peel compared to the pressed juice, while the content of free sugars, especially sucrose, was notably low. The hesperidin content in the peel is about seven times higher than in the pressed juice, and the high hesperidin content suggests that unripe citrus peel could be a potential functional material. Furthermore, we observed that the peel exhibited superior efficacy in suppressing the differentiation of adipocytes compared to pressed juice, and this efficacy increased in a concentration-dependent manner and was associated with the inhibition of the expression of adipogenesis-related genes. Therefore, unripe citrus demonstrates an anti-obesity effect by inhibiting adipogenesis and adipocyte differentiation, with the peel showing a more pronounced effect compared to pressed juice. Through this finding, our research provides optimal conditions for exploring and commercializing unripe citrus as functional materials for anti-obesity effects.

## Figures and Tables

**Table 1:** Primer sequence for RT-PCR.

Gene	Accession No.		Sequence
*Pparγ*	AB644275	Forward	5'-TTTTCAAGGGTGCCAGTTTC-3'
		Reverse	3'-AATCCTTGGCCCTCTGAGAT-5'
*C/ebpα*	NM_001287523	Forward	5'-TTACAACAGGCCAGGTTTCC-3'
		Reverse	3'-GGCTGGCGACATACAGATCA-5'
*β-actin*	EF095208	Forward	5'-GACAACGGCTCCGGCATGTGCAAAG-3'
		Reverse	3'-TTCACGGTTGGCCTTAGGGTTCAG-5'

**Table 2:** General ingredients of unripe citrus peel and pressed juice. Unit (%)

Ingredient		Unripe citrus peel (Dried)	Unripe citrus pressed juice
Moisture		16.13 ± 0.15	94.59 ± 0.08
Dry matter	Crude protein	9.91 ± 0.01	5.73 ± 0.52
	Crude fat	1.29 ± 0.19	4.16 ± 0.13
	Crude fiber	14.06 ± 0.35	0.09 ± 0.13
	Crude ash	3.87 ± 0.03	3.97 ± 0.39

**Table 3:** pH and soluble solids (Brix) of unripe citrus peel and pressed juice.

Item	Unripe citrus peel extract (10%)		Unripe citrus pressed juice	
	None	Heat-treated	None	Heat-treated
pH	3.49 ± 0.01	3.47 ± 0.02	2.72 ± 0.01	2.76 ± 0.00
Brix (%)	3.2 ± 0.0	5.1 ± 0.0	6.0 ± 0.0	6.4 ± 0.0

**Table 4:** The content of hesperidin and its derivatives in unripe citrus peel and pressed juice. Unit (mg/kg wet basis)

Compound	Unripe citrus peel		Unripe citrus pressed juice	
	None	Heat-treated	None	Heat-treated
Hesperidin	448.8 ± 12.0	3157.6 ± 122.3	76.8 ± 2.3	455.5 ± 54.5
Hesperetin-7-O-glucoside	627.5 ± 23.2	766.7 ± 16.7	46.2 ± 0.4	75.2 ± 5.1
Hesperetin	ND^a^	ND	ND	ND

^a^ND means not detected.
